# Further Insight into the Toxic Effects of Estragole, Methyleugenol, Myristicin, and Elemicin in V79 Cells

**DOI:** 10.3390/molecules31213923

**Published:** 2026-11-01

**Authors:** Lucas Wittek, Marlies Sagmeister, Anja Köllner, Mario E. Götz, Bernd Schäfer, Andreas Eisenreich

**Affiliations:** Department of Food and Feed Safety in the Food Chain, German Federal Institute for Risk Assessment, Max-Dohrn-Straße 8-10, 10589 Berlin, Germanybernd.schaefer@bfr.bund.de (B.S.)

**Keywords:** alkenylbenzenes, estragole, methyleugenol, myristicin, elemicin, cytotoxicity, DNA integrity

## Abstract

Alkenylbenzenes occur naturally in various herbs and spices, such as basil and nutmeg, and are therefore also found in various foods made from them, such as basil-containing pesto and plant food supplements. The alkenylbenzenes estragole and methyleugenol are well studied and known to induce severe toxic effects. In contrast, structurally closely related myristicin and elemicin are less well studied regarding their toxic potential. In order to address existing data gaps, we here studied the impact of estragole, methyleugenol, myristicin, and elemicin on cytotoxicity (determined via WST-1 assay) and on deoxyribonucleic acid (DNA) integrity (assessed by measurement of histone H2AX phosphorylation) in V79 cells in a comparative manner. We found that estragole, methyleugenol, myristicin, and elemicin induced cytotoxicity and impaired DNA integrity in a concentration-dependent manner in the absence and in the presence of microsomal S9 rat liver cell extract. The intensity of these effects varied among the tested compounds. The most intense effects on cytotoxicity and H2AX phosphorylation were observed for estragole and myristicin at 500 µM. Elemicin also significantly increased H2AX phosphorylation, whereas its cytotoxic impact was rather mild. Finally, methyleugenol induced only moderate effects on cytotoxicity and H2AX phosphorylation in V79 cells. Based on our findings, we concluded that the alkenylbenzenes examined in this study differ in their cytotoxic potential, which may be due to their structural properties. Moreover, mechanistic analysis assessing H2AX phosphorylation showed the tested alkenylbenzenes to induce DNA-destabilizing processes in V79 cells, which are indicative for potential genotoxic or proapoptotic effects in this experimental setting.

## 1. Introduction

Alkenylbenzenes are food-borne compounds naturally occurring in different herbs and spices, such as basil, nutmeg, and fennel seeds [1]. Particularly high concentrations can be found in essential oils or other food products derived from these herbs and spices, such as basil-containing pesto, fennel tea, and plant food supplements [2,3].

**Figure 1:**
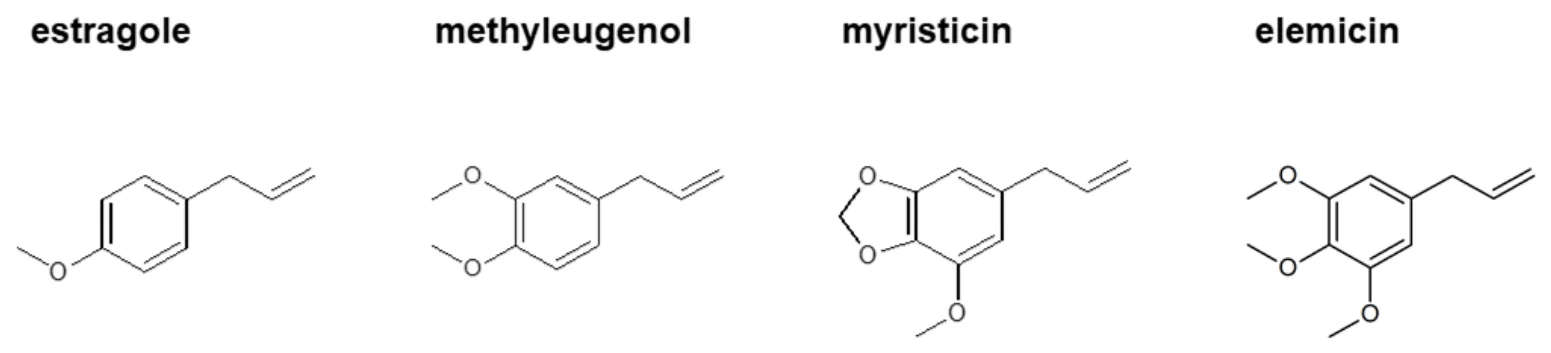
Depiction of the structural formulas of the alkenylbenzenes estragole (Chemical Abstracts Service number (CAS No.): 140-67-0), methyleugenol (CAS No.: 93-15-2), myristicin (CAS No.: 607-91-0), and elemicin (CAS No.: 487-11-6).

Some alkenylbenzenes, such as estragole and methyleugenol are well studied [2]. Based on the available scientific data they are known to induce severe toxic effects, such as genotoxicity and carcinogenicity [4,5]. In contrast to estragole and methyleugenol, the structurally closely related alkenylbenzenes myristicin and elemicin are less well studied regarding their potential toxic properties, so far [2,6]. However, given their structural similarity (Figure 1), it can be assumed that they may exhibit a toxicological potential comparable to that of estragole and methyleugenol, respectively [6].

Results of some studies indicate myristicin and elemicin to induce cellular toxicity as well as potential genotoxic effects [7,8,9]. In this context, we recently published the results of a comparative study on the potentially toxicologically relevant effects of estragole, methyleugenol, myristicin, and elemicin in V79 cells. Overall, our results did not rule out possible genotoxic properties of the abovementioned substances. In addition, our data showed concentration-dependent effects of the studied compounds on cell viability in vitro [10].

Now, we here set out to further investigate the toxic effects of estragole, methyleugenol, myristicin, and elemicin in a comparative manner. In this regard, we determined the effects of these compounds on cell viability via cytotoxicity assessment in V79 cells in the absence or the presence of an exogenous metabolizing system (S9). In order to get further mechanistic information, we also studied alkenylbenzene-induced effects on deoxyribonucleic acid (DNA) integrity in this experimental setting. This was determined via measurement of the phosphorylation at Ser139 of the subtype of histone H2A, referred to as H2AX, which is a widely accepted and sensitive indicator test for DNA double-strand-break (DSB) formation [11].

## 2. Results

We studied the impact of 1–500 µM estragole, methyleugenol, myristicin, and elemicin on cytotoxicity (measured via WST-1 assay) and DNA integrity in response to cellular stress (determined via the phosphorylation of histone H2AX (γH2AX), which was assessed as a surrogate marker for double-strand breaks in DNA) in V79 cells in a comparative manner.

### 2.1. Cytotoxic Effects of Alkenylbenzenes in V79 Cells

**Figure 2:**
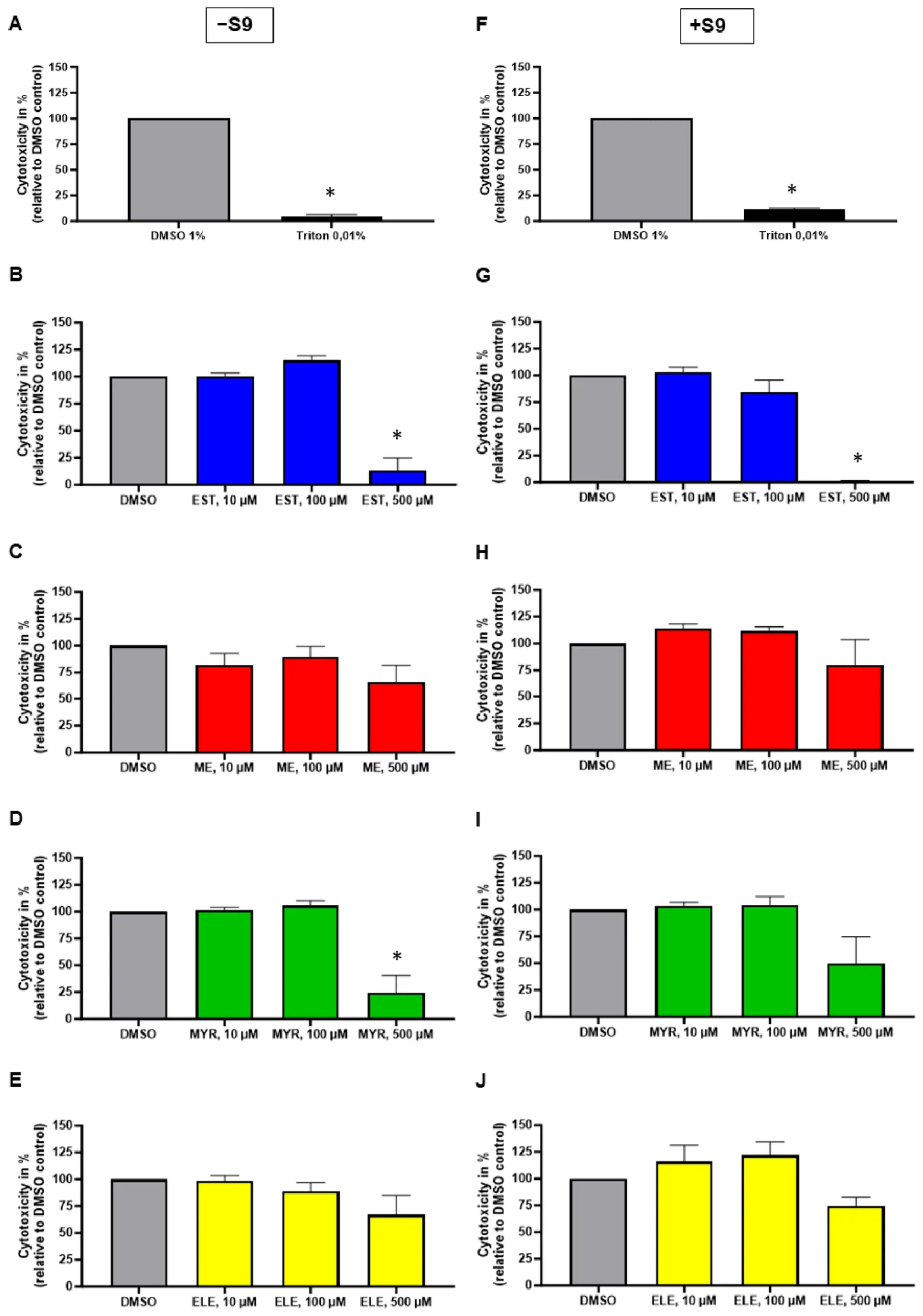
Impact of alkenylbenzenes on cytotoxicity. Shown are the results of the WST-1 assay performed in V79 cells treated with 10–500 µM estragole (EST), methyleugenol (ME), myristicin (MYR), or elemicin (ELE), (**A**–**E**) in the absence (−S9) or (**F**–**J**) in the presence of exogenous metabolization (+S9). Cells were exposed for 4 h and sampled after 24 h. Results are depicted as cytotoxicity in relation to DMSO-treated cells. The positive control was 0.01% Triton-X100 (triton 0.01%), and 1% DMSO was used as the negative control. The mean ± SEM of at least 6 independent experiments is shown. (*) = *p* < 0.05.

The influence of alkenylbenzenes on cytotoxicity was determined in V79 cells after 24 h, including 4 h exposure in the absence (Figure 2A–E) or presence (Figure 2F–J) of S9.

In the absence of an exogenous metabolizing system (S9), treatment of V79 cells with 0.01% Triton-X100 (used as a positive control) induced a significant cytotoxic effect after 24 h (Figure 2A). Exposure of cells to 10 and 100 µM estragole had no impact, whereas 500 µM estragole led to significant cytotoxicity (Figure 2B). Additionally, 10–500 µM methyleugenol showed no significant influence in the WST-1 assay (Figure 2C). Treatment of cells with 10 and 100 µM myristicin had also no effect, whereas 500 µM myristicin significantly induced cytotoxicity after 24 h (Figure 2D). In contrast, exposure of V79 cells to elemicin had no significant impact on cytotoxicity in all tested dose groups (10–500 µM; Figure 2E).

In the presence of S9, 0.01% Triton-X100 (positive control) significantly affected cytotoxicity in V79 cells after 24 h (Figure 2F). Treatment of cells to 10 or 100 µM estragole showed no effect in the WST-1 assay, whereas 500 µM estragole significantly induced cytotoxicity after 24 h (Figure 2G). Methyleugenol showed no significant influence on cytotoxicity in all tested dose groups (10–500 µM; Figure 2H). Exposure of cells to 10 and 100 µM myristicin showed no effect, whereas 500 µM myristicin led to a slight—but not significant—cytotoxic influence (Figure 2I). Finally, 10–500 µM elemicin had no significant impact on cytotoxicity in V79 cells after 24 h (Figure 2J).

### 2.2. Alkenylbenzenes Affect DNA Integrity in V79 Cells

**Figure 3:**
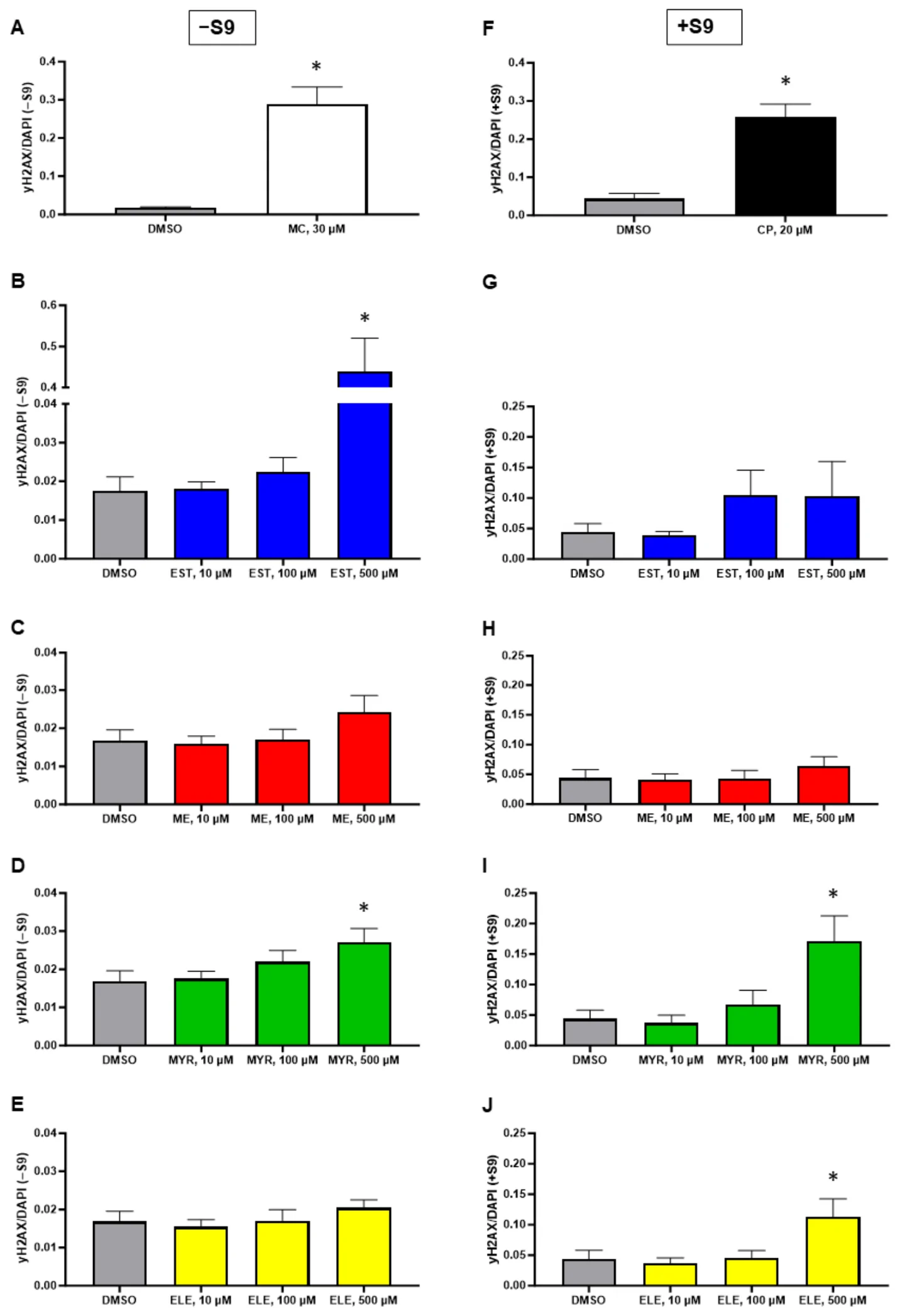
Effect of alkenylbenzenes on the histone H2AX phosphorylation (γH2AX) assessed as a surrogate marker for DNA double-strand breaks (DSB) in V79 cells. Depicted is the ratio of γH2AX normalized to DAPI-stained nuclei (DAPI). Cells were exposed for 4 h to 1–500 µM estragole (EST), methyleugenol (ME), myristicin (MYR), or elemicin (ELE), (**A**–**E**) in the absence (−S9) or (**F**–**J**) in the presence of exogenous metabolization (+S9) and sampled after 24 h. The positive controls were 30 µM mitomycin C (MC; −S9) and 20 µM cyclophosphamide (CP; +S9), and 1% DMSO was used as the negative control. The mean ± SEM of at least 6 independent experiments is shown. (*) = *p* < 0.05.

Possible effects of alkenylbenzenes on the phosphorylation at Ser139 of the histone H2AX, in response to DSB formation, were determined in V79 cells after 24 h, including 4 h exposure in the absence (Figure 3A–E) or presence (Figure 3F–J) of S9, followed by a recovery phase of 20 h.

In the absence of S9, treatment of cells with 30 µM mitomycin C (MC, used as a positive control for a DNA-damaging agent that does not require metabolic activation) significantly increased γH2AX formation after 24 h (Figure 3A). Treatment of V79 cells with 10–100 µM estragole had no impact, whereas 500 µM estragole led to a significant induction of γH2AX frequency (Figure 3B). Additionally, 10–500 µM methyleugenol showed no significant influence on the formation of γH2AX (Figure 3C). Exposure of cells to myristicin led to a slight but concentration-dependent increase in γH2AX frequency, which was significant at 500 µM (Figure 3D). Treatment of cells with elemicin had no significant impact on cytotoxicity in all tested dose groups in the absence of S9 (Figure 3E).

In the presence of S9, cyclophosphamide (CP, used as a metabolically activated positive control) resulted in a significant increase of γH2AX formation, demonstrating sufficient effectiveness of the exogenous metabolic activation system (Figure 3F). Exposure of V79 cells to 10 µM estragole showed no effect, whereas 100 µM and 500 µM estragole led to a slight but not significant induction of the γH2AX frequency after 24 h (Figure 3G). Methyleugenol had no significant influence on γH2AX formation in all tested dose groups (10–500 µM; Figure 3H). In contrast, myristicin increased the γH2AX frequency in a dose-dependent manner, with this effect becoming significant at 500 µM (Figure 3I). Finally, 10 µM and 100 µM elemicin had no significant influence, whereas 500 µM elemicin significantly increased the γH2AX formation in V79 cells after 24 h (Figure 3J).

## 3. Discussion

In contrast to the well-known toxic compounds estragole and methyleugenol, little information is available on the potential toxicological properties of the structurally closely related alkenylbenzenes myristicin and elemicin [2,6]. However, there is some evidence indicating that these alkenylbenzenes may cause both cellular toxicity and genotoxic effects [9,12,13]. We recently published a comparative analysis of estragole, methyleugenol, myristicin, and elemicin with regard to their genotoxic potential in V79 cells with or without metabolic activation [10]. The data published in this context not only provided information on the role of these alkenylbenzenes in micronucleus formation, but also indicated proapoptotic effects triggered by them. Here, we set out to further elucidate the effects of estragole and methyleugenol compared to myristicin and elemicin, especially on cell viability and DNA integrity in the same experimental setting.

### 3.1. Cytotoxic Effects of Estragole, Methyleugenol, Myristicin and Elemicin in V79 Cells

In our study, we found that low concentrations (10–100 µM) of all the alkenylbenzenes tested had no significant effect on cell viability (Figure 2), either in the absence or in the presence of an exogenous metabolic system (S9). However, 500 µM estragole or the structurally similar compound myristicin clearly induced cytotoxicity in both experimental settings. Treatment of cells with 500 µM of the structurally related compounds methyleugenol or elemicin, respectively, led to a rather slight cytotoxic effect in the absence as well as in the presence of S9. In contrast to estragole and myristicin, however, this effect was not significant in V79 cells treated with methyleugenol or elemicin (Figure 2). This is in line with the findings of other groups [14,15]. In 2012, Martins et al. demonstrated that low estragole concentration (10 to <500 µM) had no influence on cell viability, whereas high doses of 500–3500 µM induced significant cytotoxicity in V79 cells in a concentration-dependent manner [14]. Moreover, Groh and colleagues showed in 2012 that low doses (10–100 µM) of methyleugenol and some selected metabolites induce no or only minor cytotoxic effects in V79 cells after 24–72 h [15]. Further substantiating our results, comparable effects were also observed in other experimental settings [16,17]. Howes and colleagues demonstrated in 1990 that low concentrations (1–100 µM) of estragole and methyleugenol had no influence on cell viability of primary rat hepatocytes, determined via a lactate dehydrogenase assay after 17 h [17]. In contrast, high concentrations (500–10,000 µM) of these compounds clearly reduced cell viability in this experimental setting. In line with this, Chan and Caldwell showed in 1992 that 1–100 µM estragole and methyleugenol had no effects on the viability of primary rat hepatocytes, whereas higher doses starting at 500 µM led to reduced cell viability after 17 h [16].

Information regarding the cytotoxic potential of elemicin or myristicin is rather sparse. However, in 1991 Hasheminejad and Caldwell published data showing that low concentrations (<1000 µM) of elemicin had no influence on cell viability of cultured rat hepatocytes in vitro, whereas 1000–10,000 µM decreased cell viability after 17 h in a dose-dependent manner [18]. Moreover, they found that low doses (<500 µM) of myristicin had also no impact on rat hepatocyte viability, whereas concentrations of 500–10,000 µM significantly reduced cell viability in this setting. This is in line with our findings, also showing no effect of 10–500 µM elemicin or of 10–100 µM myristicin on cell viability, whereas cytotoxicity was clearly increased at 500 µM myristicin in the absence and in the presence of S9 (Figure 2). This is further substantiated by earlier published data in a comparable experimental setting, showing that 10–500 µM elemicin as well as 10–100 µM myristicin had no influence on viability of V79 cells in the context of a micronucleus assay [10]. In contrast, treatment of cells with 500 µM myristicin significantly reduced V79 cell viability after 24 h [10].

Together these data suggest that estragole, methyleugenol, myristicin, and elemicin differentially affect cytotoxicity in V79 cells, in the absence as well as in the presence of S9. The observed cytotoxic effects were most pronounced for the treatment of cells with estragole and the structurally related myristicin. Rather mild effects were observed for methyleugenol and the structurally closely related substance elemicin in this study.

### 3.2. Estragole, Methyleugenol, Myristicin, and Elemicin Affect DNA Integrity in V79 Cells

In a recently published study, we showed that treatment of V79 cells with estragole, methyleugenol, myristicin, and elemicin led to increased cell death and that this was associated with proapoptotic processes [10]. In line with our findings, different groups also showed that alkenylbenzenes, such as methyleugenol or myristicin, induced apoptosis in human cancer cells [13,19,20]. Moreover, myristicin and methyleugenol were demonstrated to induce apoptosis in human leukemia cells and hepatocytes, respectively, through mitochondrial effects [19,21]. Beside proapoptotic effects, we also showed in this earlier published study that treatment of V79 cells with estragole, methyleugenol, myristicin, or elemicin was associated with the occurrence of genotoxic effects, as determined via micronucleus formation—at least in the groups receiving the highest dose of 500 µM [10]. This is in line with data published by others, also demonstrating genotoxic effects of alkenylbenzenes, such as estragole or myristicin, in vitro as well as in vivo [9,22].

Apoptosis is often directly linked to DNA damage, which is triggered, among other things, by double-strand breaks in DNA [23,24]. In this context, DNA damage leads to mitochondrial dysfunction, which triggers further proapoptotic processes consequently leading to cell death [25]. Moreover, it is well known that different metabolic pathways of alkenylbenzenes lead to the formation of reactive intermediates capable of causing genotoxic effects by forming DNA adducts [6,26]. If remaining unrepaired, DNA adducts may result in impairment of DNA replication, releasing nicks. At positions where two complementary strands of the double helix of DNA are damaged simultaneously in locations close to each other, DSB may occur [27]. It is reported that a single unrepaired DSB is sufficient for the induction of the cell-death process [28]. Together, this information suggests a possible mechanistic link between the proapoptotic as well as the genotoxic potential of the alkenylbenzenes, which arises through DNA damage and consequently the induction of DSB.

Since the phosphorylation at Ser139 of the histone H2AX (called γH2AX) was observed in response to DSB formation in mammalian cells [29], γH2AX is considered a suitable biomarker for DNA integrity [30]. Therefore, γH2AX assessment is often used as an indicator test for the formation of DSB.

To shed more light on the underlying mechanisms of the earlier described proapoptotic and genotoxic potential of estragole, methyleugenol, myristicin, and elemicin [10], we here assessed the impact of these alkenylbenzenes on DNA integrity in V79 cells via measurement of γH2AX.

Our results showed that all tested alkenylbenzenes increased phosphorylation of H2AX in a concentration-dependent manner, both in the absence and in the presence of S9 (Figure 3), even though these effects varied in intensity. In this context, these effects were most pronounced when cells were treated with high concentrations (500 µM) of estragole, myristicin, and elemicin, whereas treatment of cells with methyleugenol led to rather slight effects regarding γH2AX induction. These findings are in line with the results of others [14,21]. In 2012, Martins et al. demonstrated that treatment of the Chinese hamster ovary cell lines AA8 and EM9 with estragole induced DNA damage and that this was also associated with cell apoptosis as well as with increased DNA adduct formation, indicating its genotoxic potential in vitro [14]. Further substantiating our results and highlighting the influence of hepatic metabolism of alkenylbenzenes, Carlsson et al. showed in 2022 that hydroxylated metabolites of methyleugenol induce γH2AX in HepG2 liver cells and transgenic metabolically competent V79 (CS) cells [21]. Moreover, this was associated with increased cell death in V79 cells and mitochondrially triggered apoptosis of HepG2 liver cells as well as with elevated DNA adduct formation in both cell lines [21]. Furthermore, DNA-damaging effects were also demonstrated for estragole, methyleugenol, myristicin, and elemicin in vivo in a turkey egg fetal liver model leading to DNA adducts [8]. In this context, the authors concluded that their results provide further evidence for the genotoxic potential—and possible mutagenic effects mediated via DNA-adduct formation—of these alkenylbenzenes. Finally, Ackermann et al. recently showed 10–2000 µM estragole to significantly induce the formation of DNA adducts in a cytochrome P450 (Cyp)1A2-overexpressing human liver cell line (HepG2) [31]. This was associated with significantly increased γH2AX formation in these cells at a concentration of 100–1000 µM. Further, they demonstrated that the estragole metabolite 1′-hydroxyestragole also induced the generation of DNA adducts at 1–35 µM and γH2AX formation at a concentration of 1–50 µM in wildtype HepG2 cells. Additionally, they found 25–50 µM 1′-hydroxyestragole to induce the formation of micronuclei in HepG2 in a dose-dependent manner [31]. Although no significant effect on micronucleus formation was observed in this experimental setup in Cyp1A2-overexpressing HepG2 cells treated with estragole, these results nevertheless suggest that—at least—metabolically activated estragole exhibits a genotoxic potential in vitro. Further substantiating its genotoxic potential, Ackermann and colleagues recently showed that estragole also induced the generation of DNA adducts in human liver tissue [26].

Together, these findings suggest that estragole, methyleugenol, myristicin, and elemicin differentially affect DNA integrity, as determined via H2AX phosphorylation measurement in V79 cells, in the absence as well as in the presence of S9. The observed effects were most pronounced for treatment of cells with estragole, structurally related myristicin and with elemicin in the presence of S9. Rather moderate effects were induced by methyleugenol in this experimental setting.

### 3.3. Estragole, Methyleugenol, Myristicin and Elemicin Differentially Induce Toxic Effects in V79 Cells

Overall, our results and the discussion of further findings published by other groups show that the alkenylbenzenes examined in this study differ in their cytotoxic potential (estragole > myristicin > elemicin > methyleugenol) in V79 cells in the absence as well as in the presence of an exogenous metabolizing system. These effects may be related to the structural properties of the tested compounds. Moreover, cytotoxicity was associated with DNA-destabilizing effects in this context. This—in turn—may be mechanistically involved in the alkenylbenzene-induced proapoptotic processes and potential genotoxic effects observed in an earlier study performed under the same experimental conditions [10].

Increased phosphorylation of H2AX resulting from the induction of DSB can be associated not only with apoptosis but also with other cellular processes, such as replication stress and genotoxicity [30,32]. Therefore, we cannot exclude that here observed increased H2AX phosphorylation—especially at high doses of estragole, myristicin and elemicin—was induced by genotoxic effects of these compounds, as suggested for estragole and methyleugenol by others, too [14,21]. Endogenous metabolic activation of alkenylbenzenes has been shown to be crucial in the context of genotoxicity induced by these compounds [6,33]. In this context, the lack of metabolic capacity in V79 cells, the use of rat S9 as an artificial tool to compensate for this deficiency, and potential effects of chemical impurities (e.g., in myristicin) represent methodological limitations whose impacts on the study results are difficult to assess. Therefore, in future studies, it would be useful to conduct investigations in more sophisticated experimental systems that do not have such limitations (e.g., those possessing endogenous metabolic capacity, such as human hepatic cells). Such information, in particular, would be highly relevant for a more reliable risk assessment of these substances with regard to human health in the context of consumption of foods containing alkenylbenzenes [2,34]. Therefore, further studies are needed to elucidate the toxicological effects of alkenylbenzenes such as estragole, methyleugenol, myristicin and elemicin, particularly with regard to their genotoxic profiles, including mutagenicity as consequences of DNA adduct formation and impairment of DNA repair mechanisms. In this context, potential differences and kinetics of their endogenous metabolic activations would have to be addressed in future studies. This could help to fill relevant data gaps with the aim to further reduce considerable uncertainties currently impeding the evaluation of adverse health effects potentially caused by these alkenylbenzenes.

## 4. Conclusions

In summary, the findings reported and discussed in this manuscript indicate that estragole, methyleugenol, myristicin, and elemicin may differentially affect cytotoxicity and DNA integrity in V79 cells. However, there are important methodological limitations—especially regarding the lack of endogenous metabolic capacity—of this as well as other standard in vitro test settings. This emphasizes the need to further develop existing in vitro toxicity testing systems that do not rely on exogenous metabolizing systems (S9) in order to increase specificity and sensitivity to substances that are metabolically activated to highly toxic compounds.

## 5. Materials and Methods

### 5.1. Cell Culture

V79 cells (Chinese hamster lung fibroblasts, obtained from Dr. Hansruedi Glatt, German Institute of Human Nutrition, Nuthetal, Germany) were cultured in Dulbecco’s Modified Eagle Medium (DMEM; Pan-Biotech, Aidenbach, Germany), containing 10% fetal bovine serum (FBS; Pan-Biotech, Aidenbach, Germany) and 1% penicillin/streptomycin (Capricorn Scientific, Ebsdorfergrund, Germany) at 37 °C in a humidified incubator (5% CO_2_, 95% air). Cells were treated with 10, 100, and 500 µM estragole (Chemical Abstracts Service number (CAS#) 140-67-0, purity = 98%; item number: A29208), methyleugenol (CAS# 93-15-2, purity > 98%; item number: 04607), myristicin (CAS# 607-91-0, purity ≥ 85%; item number: M-9411), and elemicin (CAS# 487-11-6, purity = 98%; item number: OR40002), all provided by Sigma-Aldrich, Taufkirchen, Germany. All test substances and positive controls were solved in dimethyl sulfoxide (DMSO; Merck, Darmstadt, Germany). Treatment was performed for 4 h in the presence (+) or in the absence (−) of 10% S9 mix (corresponding to approximately 0.5 mg/ml total protein from S9), consisting of 2.85 mL liver S9 (supernatant fraction from homogenized liver after centrifugation at 9000× *g*) from 5-to-6-week-old male Sprague Dawley rats induced with phenobarbital and 5,6-benzoflavone (Trinova Biochem GmbH, Gießen, Germany), 4.65 mL sterile water, and 15 mL co-factor solution (8.0 mM MgCl_2_/6H_2_O (Supelco, St. Louis, MO, USA), 33.0 mM KCl (Merck, Darmstadt, Germany), 67.2 mM Na_2_HPO_4_/2H_2_O (Merck, Darmstadt, Germany), 15.8 mM NaH_2_PO_4_/2H_2_O (Merck, Darmstadt, Germany), 5.62 mM glucose-6-phosphate (Carl Roth, Karlsruhe, Germany), 4.1 mM NADPH (Carl Roth, Karlsruhe, Germany), and 4.3 mM NADH (Carl Roth, Karlsruhe, Germany)). After that, medium was replaced by fresh medium (without S9 mix) for a further 20 h (recovery phase). As described earlier [10], the experimental setup—including dose groups for alkenylbenzenes and controls as well as incubation times—was determined in preliminary tests.

### 5.2. WST-1 (Water Soluble Tetrazolium-1) Cytotoxicity Assay

Next, 15,000 cells/well were seeded in 96-well plates for 24 h, and the cells were treated for 4 h with 10, 100, and 500 µM estragole, methyleugenol, myristicin, or elemicin, followed by a recovery period of a further 20 h. After that, 0.5 mg/ml WST-1 reagent (Roche Diagnostics GmbH, Mannheim, Germany) was added and cells were incubated for 1 h at 37 °C. Finally, light absorption of the samples was measured with a Tecan infinite M200 PRO microplate reader (Tecan Group Ltd., Männedorf, Switzerland) according to the manufacturer’s recommendation. As the positive control for cytotoxicity measurement, 0.01% Triton X-100 (AppliChem GmbH, Darmstadt, Germany) was used; 1% DMSO was used as the negative control, and the results are depicted as cytotoxicity in relation to DMSO (*n* = 6).

### 5.3. Histone H2AX Phosphorylation (γH2AX) Assay

The phosphorylation of the histone H2AX (γH2AX) was assessed as a surrogate marker for double-strand breaks in DNA. First, 2500 cells/well were seeded in 96-well plates for 24 h and then treated for 4 h with 10, 100, and 500 µM estragole, methyleugenol, myristicin, or elemicin, followed by a recovery period of another 20 h. Then, cells were washed 3 times with phosphate-buffered saline (PBS) and fixed with 95% methanol for 30 min at room temperature. After that, the methanol was removed and the cells were washed with PBS + 0.05% Tween (PBS-T) for 2 min at room temperature, and blocked using 1% bovine serum albumin (BSA) solved in PBS-T for 1 h at room temperature. After washing with PBS-T, samples were incubated with the primary antibody (anti-phospho-Histone H2A.X (Ser139) antibody, mouse clone JBW301; EMD Millipore Corporation, Burlington, MA, USA; 1:500 in 1% BSA/PBS-T) for 1 h at room temperature. Then, wells were washed 3 times with PBS-T and incubated with the goat anti-mouse IgG (H + L) cross-adsorbed secondary antibody Alexa Fluor™ 647 (Life Technologies GmbH, Darmstadt, Germany; 1:400 in 1% BSA/PBS-T) for 1 h at room temperature. After washing (3 times with PBS-T), staining of nuclei was done using 4′,6-diamidino-2-phenylindole (DAPI, 1:1000/PBS) purchased from Life Technologies GmbH (Darmstadt, Germany) for a further 30 min at room temperature. Finally, fluorescence intensity was determined using a Zeiss Cell Discoverer 7 high-content screening microscope (Zeiss, Jena, Germany). As controls for DNA-damaging substances, 20 µM cyclophosphamide (CP, positive control in presence of S9; Sigma-Aldrich) or 30 µM mitomycin C (MC, positive control in the absence of S9; Sigma-Aldrich) were used, and 1% DMSO was used as the negative control (*n* = 6).

### 5.4. Statistical Analysis

All data were expressed as mean ± SEM. Data were analyzed by one-way analysis of variance (ANOVA). A probability value (*p*) < 0.05 was regarded as significant. Statistical analyses were done using GraphPad Prism, version 10.1.2 (GraphPad Software, Boston, MA, USA).

## Data Availability

The raw data supporting the conclusions of this article will be made available by the authors on request.
